# Comparative Analysis of Measures of Viral Reservoirs in HIV-1 Eradication Studies

**DOI:** 10.1371/journal.ppat.1003174

**Published:** 2013-02-14

**Authors:** Susanne Eriksson, Erin H. Graf, Viktor Dahl, Matthew C. Strain, Steven A. Yukl, Elena S. Lysenko, Ronald J. Bosch, Jun Lai, Stanley Chioma, Fatemeh Emad, Mohamed Abdel-Mohsen, Rebecca Hoh, Frederick Hecht, Peter Hunt, Ma Somsouk, Joseph Wong, Rowena Johnston, Robert F. Siliciano, Douglas D. Richman, Una O'Doherty, Sarah Palmer, Steven G. Deeks, Janet D. Siliciano

**Affiliations:** 1 Department of Diagnostics and Vaccinology, Swedish Institute for Communicable Diseases and Department of Microbiology, Tumor and Cell Biology, Karolinska Institutet, Solna, Sweden; 2 Department of Pathology and Laboratory Medicine, University of Pennsylvania, Philadelphia, Pennsylvania, United States of America; 3 University of California San Diego, La Jolla, California and Veterans Affairs San Diego Healthcare System, San Diego, California, United States of America; 4 San Francisco VA Medical Center, San Francisco, California, United States of America; 5 Department of Medicine, University of California, San Francisco, San Francisco, California, United States of America; 6 Department of Biostatistics, Harvard School of Public Health, Boston, Massachusetts, United States of America; 7 Department of Medicine Johns Hopkins University School of Medicine, Baltimore, Maryland, United States of America; 8 amfAR, The Foundation for AIDS Research, New York, New York, United States of America; 9 Howard Hughes Medical Institute, Baltimore, Maryland, United States of America; National Institutes of Health, National Institute of Allergy and Infectious Diseases, United States of America

## Abstract

HIV-1 reservoirs preclude virus eradication in patients receiving highly active antiretroviral therapy (HAART). The best characterized reservoir is a small, difficult-to-quantify pool of resting memory CD4^+^ T cells carrying latent but replication-competent viral genomes. Because strategies targeting this latent reservoir are now being tested in clinical trials, well-validated high-throughput assays that quantify this reservoir are urgently needed. Here we compare eleven different approaches for quantitating persistent HIV-1 in 30 patients on HAART, using the original viral outgrowth assay for resting CD4^+^ T cells carrying inducible, replication-competent viral genomes as a standard for comparison. PCR-based assays for cells containing HIV-1 DNA gave infected cell frequencies at least 2 logs higher than the viral outgrowth assay, even in subjects who started HAART during acute/early infection. This difference may reflect defective viral genomes. The ratio of infected cell frequencies determined by viral outgrowth and PCR-based assays varied dramatically between patients. Although strong correlations with the viral outgrowth assay could not be formally excluded for most assays, correlations achieved statistical significance only for integrated HIV-1 DNA in peripheral blood mononuclear cells and HIV-1 RNA/DNA ratio in rectal CD4^+^ T cells. Residual viremia was below the limit of detection in many subjects and did not correlate with the viral outgrowth assays. The dramatic differences in infected cell frequencies and the lack of a precise correlation between culture and PCR-based assays raise the possibility that the successful clearance of latently infected cells may be masked by a larger and variable pool of cells with defective proviruses. These defective proviruses are detected by PCR but may not be affected by reactivation strategies and may not require eradication to accomplish an effective cure. A molecular understanding of the discrepancy between infected cell frequencies measured by viral outgrowth versus PCR assays is an urgent priority in HIV-1 cure research.

## Author Summary

Efforts to cure HIV-1 infection have focused on a small pool of CD4^+^ T cells that carry viral genetic information in a latent form. These cells persist even in patients on optimal antiretroviral therapy. Novel therapeutic strategies targeting latently infected cells are being developed, and therefore practical assays for measuring latently infected cells are urgently needed. These cells were discovered using a virus culture assay in which the cells are induced to release virus particles that are then expanded in culture. This assay is difficult, time-consuming, and expensive. Here we evaluate alternative approaches for measuring persistent HIV-1, all of which rely on the detection of viral genetic information using the polymerase chain reaction (PCR). None of the PCR-based assays correlated precisely with the virus culture assay. The fundamental problem is that infected cell frequencies determined by PCR are at least 2 logs higher than frequencies determined by the culture assay. Much of this difference may be due to cells carrying defective forms of the virus. These cells may not be eliminated by strategies designed to target latently infected cells. In this situation, successful clearance of latently infected cells might be masked by a large unchanging pool of cells carrying defective HIV-1.

## Introduction

Treatment of HIV-1 infection with highly active antiretroviral therapy (HAART) can reduce plasma HIV-1 RNA levels in treated patients to below the detection limit of clinical assays (50 copies of HIV-1 RNA/ml) [1]–[3]. The effective suppression of viremia initially encouraged hopes that the virus could be eradicated with two to three years of HAART [3]. However, a latent form of HIV-1 infection persists *in vivo* [4], [5]. A small fraction of resting memory CD4^+^ T cells carry integrated viral genomes. These cells do not produce virus particles while in the resting state, but can give rise to replication-competent virus following cellular activation [4], [5]. These latently infected cells are rare but stable, even in patients on prolonged HAART [6]–[11]. Interruption of HAART leads to a rebound in viremia [12], [13], typically from an archival variant [14]. The latent reservoir is widely recognized as the major barrier to HIV-1 eradication [15].

Strategies aimed at reactivating latent virus and thereby accelerating the clearance of the latent reservoir are now in advanced pre-clinical testing or early clinical trials [15]. Approaches for the reactivation of latent HIV-1 include T cell activating cytokines [16]–[19], T cell receptor and T cell receptor signaling pathway agonists [20]–[24], histone deacetylase inhibitors [25]–[27], DNA methylase inhibitors [28], [29], and compounds like 5-hydroxynaphthalene-1,4-dione [30] and disulfiram [31]. A single dose of the histone deacetylase inhibitor suberoylanilide hydroxamic acid (SAHA) has recently been shown to increase cell-associated HIV-1 RNA in CD4^+^ T cells from patients on HAART [32].

A major question for current and future trials of eradication strategies is how to evaluate the effectiveness of the interventions. The principal approach for quantifying HIV-1 persistence during HAART is a viral outgrowth assay performed on highly purified resting CD4^+^ T cells. These cells do not produce virus without stimulation [4]. In the assay, limiting dilutions of resting CD4^+^ T cells are stimulated with the mitogen phytohemagglutinin (PHA) or with anti-CD3 plus anti-CD28 antibodies in the presence of irradiated allogeneic peripheral blood mononuclear cells (PBMC) [5], [6], [9], [10], [33]. These stimuli induce global T cell activation, which reverses latency at least in a fraction of cells carrying integrated HIV-1 genomes. The viruses released from these cells are expanded in CD4^+^ lymphoblasts from HIV-1-negative donors and detected after 2–3 weeks by an ELISA assay for HIV-1 p24 antigen in the supernatant. This assay detects individual latently infected cells that release replication-competent virus following cellular activation. The frequency of latently infected cells, expressed in terms of infectious units per million (IUPM) resting CD4^+^ T cells, is determined using Poisson statistics and is on the order of 0.1–10 IUPM in most patients on long term HAART. The value of this assay is that it detects cells that can, when activated, release viruses capable of robust replication. It therefore provides a minimum estimate of the frequency of latently infected cells that that must be eliminated to ensure eradication and is used here as a standard for comparison. In principle, this assay can also detect resting CD4^+^ T cells harboring labile unintegrated forms of HIV-1 DNA [34], [35], although the frequency of cells containing unintegrated DNA during HAART is low [36]–[38]. Although the viral outgrowth assay has important advantages, it is expensive and labor intensive, and it requires large amounts of blood (120–180 ml).

Alternative approaches generally involve PCR assays for HIV-1 DNA. Some of these assays distinguish between integrated proviruses and unintegrated HIV-1 DNA [39], [40]. A problem with all PCR-based assays is that they fail to distinguish between replication-competent and defective forms of the viral genome. A significant but poorly characterized proportion of infected resting CD4^+^ T cells contain proviruses that are defective, hypermutated, or silenced [41], [42]. PCR assays are now also being used to quantify HIV-1 DNA in CD4^+^ T cells from the gut associated lymphoid tissue (GALT), where the frequency of HIV-1 infection is generally higher than in the blood [43], [44]. Highly sensitive PCR methods are also now used to quantify HIV-1 RNA in cells [32]. Free virus particles are also found in the plasma of patients on HAART [45]–[47]. This residual viremia is an important indication of ongoing virus production. Several studies have shown that residual viremia is not reduced by treatment intensification [48]–[50], and thus it is likely to reflect virus production from stable reservoirs. For example, residual viremia could in part reflect virus production by latently infected cells that have become activated.

It is currently unclear which assay(s) should be used to monitor HIV-1 reservoirs in clinical trials of eradication strategies. The development of a high-throughput scalable assay to measure the latent reservoir in patients has been identified as a key priority in HIV-1 eradication research [51]. Here we present a comparative analysis of eleven different approaches for measuring for HIV-1 reservoirs in two well characterized cohorts of patients on long term HAART. The goal of the study was to determine how these assays correlate with the viral outgrowth assay. The results provide insights into how reservoirs should be evaluated in future clinical trials aimed at curing HIV-1 infection.

## Results

### Patient characteristics

The baseline characteristics of the cohort are shown in Table 1. Of 30 study participants, 10 started HAART during acute or early HIV-1 infection while the remaining 20 started HAART during chronic infection. The mean (±SD) age was 53.2±9.6 years. Patients starting therapy during acute/early infection were slightly younger (47.8±9.3 vs. 55.9±8.7 years). The majority (76.7%) of study subjects were white/non-Hispanic. The current CD4^+^ T cell counts were not significantly different between patients starting HAART during acute/early vs. chronic infection (727±287 vs. 672±144, P = 0.58). For patients starting HAART during chronic infection, the CD4 nadir was 202±138 cells/µl. The average duration of viral load suppression on HAART was 5.8±2.5 years for patients starting HAART during acute/early infection and 8.0±4.2 years for patients starting HAART during chronic infection. No patient in either cohort had a documented “blip” above 40 copies RNA/mL in the year preceding the blood draw.

**Table 1:** Patient characteristics.

Pt. ID	Acute/chronic^a^	Age	Sex	Race^b^	Time before viral load <50^c^ (months)	Time with viral load <50^d^ (months)	HAART Regimen^e^	Nadir CD4 count (cells/µl)	Current CD4 count (cells/µl)
1004	chronic	56	M	W	226	46	3TC/ETV/RAL/DRV/r	180	362
1033	chronic	52	M	W	85	48	TDF/FTC/EFV	330	636
1079	chronic	60	M	H	53	89	TDF/FTC/ATV/r	343	850
1126	chronic	64	M	W	156	45	TDF/FTC/EFV	227	715
1234	chronic	39	M	H	74	39	TDF/FTC/ATV/r	250	519
2007	chronic	60	M	W	70	159	ABC/3TC/EFV	150	825
2013	chronic	63	M	W	154	167	ABC/3TC/ATV	13	625
2021	chronic	44	M	AA	16	158	ABC/3TC/EFV	42	592
2026	chronic	57	M	W	149	152	ABC/3TC/TDF/DRV/r	120	610
2056	chronic	57	M→F	W	174	98	TDF/FTC/FPV/r	423	674
2104	chronic	65	M	W	149	164	ABC/3TC/ATV/r	180	634
2113	chronic	38	M	W	68	119	TDF/FTC/RAL	120	694
2114	chronic	70	M	W	163	161	TDF/FTC/EFV	270	792
2147	chronic	55	M	As	197	99	DDI/3TC/NVP	99	641
2177	chronic	62	M	H	178	61	ABC/TDF/EFV	520	864
2208	chronic	59	M	W	298	43	ABC/DDI/D4T/FPV	54	464
2451	chronic	45	M	W	85	46	TDF/FTC/NVP	124	964
3068	chronic	57	M	W	158	128	ABC/3TC/ETV/RAL	87	594
3178	chronic	64	M	W	280	44	TDF/FTC/RAL/DRV/r	389	775
4001	chronic	52	M→F	AA	322	58	AZT/3TC/ABC/LPV/r	138	606
2238	acute	59	M	W	3	69	TDF/FTC/RAL	538	1127
2248	acute	47	M	W	2	99	TDF/FTC/ATV/r	472	423
2263	acute	42	M	W	7	68	ABC/3TC/ATV/r	714	1052
2264	acute	61	M	W	8	86	ABC/3TC/RAL	550	1172
2277	acute	38	M	W	6	56	TDF/FTC/EFV	234	736
2286	acute	46	M	W	4	119	TDF/FTC/EFV	200	414
2418	acute	49	M	W	6	39	TDF/FTC/EFV	335	541
2420	acute	50	M	W	3	40	TDF/FTC/ATV/r	309	558
2453	acute	55	M	W	8	102	TDF/FTC/NVP	408	622
2454	acute	31	M	M	7	46	TDF/FTC/ETV	352	622

a HAART initiated during acute/early or chronic HIV-1 infection as defined in Methods.

b W, white, non-Hispanic; H, Hispanic; AA, African-American; As, Asian; M, mixed race.

c Time after infection before achieving the most proximally documented period of sustained suppression of viremia to <50 copies/ml on HAART. Patients in the acute cohort started therapy within 3 months of infection.

d Time of documented continuous suppression of viremia to <50 copies/ml on HAART.

e Drug abbreviations: 3TC, lamivudine; ABC, abacavir; ATV/r, atazanavir boosted with ritonavir; d4T, stavudine; ddI, didanosine; DRV/r, darunavir boosted with ritonavir; EFV, efavirnez; ETV, etravirine; FTC, emtricitabine; FPV, fosamprenavir; FPV/r, fosamprenavir boosted with ritonavir; NVP, nevirapine; RAL, raltegravir; TDF, tenofovir disoproxil fumarate.

### Assay characteristics

Samples were processed, split, and sent to laboratories with expertise in the assays described above. Each assay was developed independently by the relevant laboratory, with different input material, assay methodology, normalization method, statistical characteristics, and caveats. These are indicated in Table 2. The cell types analyzed and viral species detected in each assay are indicated in Table 3. Except for the single copy assay for plasma HIV-1 RNA, assay results are presented in the form of infected cell frequencies to facilitate cross-assay comparisons. However, the cell populations included and viral species detected in each assay (Table 3) must be kept in mind in interpreting these frequencies. The statistical characteristics of each assay, such as the coefficient of variation, the limit of detection, and the dynamic range (summarized in Table 2) are important considerations in choosing assays to monitor viral persistence. For example, the coefficient of variation for the viral outgrowth assay is considerably higher than that for most PCR-based assays. Statistical characteristics must also be considered in evaluating the correlations between the results of different assays because problems related assay precision, accuracy, and sensitivity can obscure correlations. Table 2 also describes the primers used for each PCR assay. Negative results for any single PCR assay on a given patient sample can reflect sequence variation in the primer binding site [47].

**Table 2:** Characteristics of assays.

Assay	Assay characteristics	Sample size	Assay input	Primers^a^	Unit of comparison	Limit of detection^b^ (LOD)	Dynamic range^c^ (logs)	Normalization method	Explanation for negative values	Ref.
Viral outgrowth	95% CI for individual determinations = ±0.7 logs based on Poisson statistics and 2 replicates/cell number; coefficient of variation = 0.95^d^	120–180 ml blood	resting CD4^+^ T cells	none	infectious units per 10^6^ (IUPM) resting CD4^+^ T cells	0.02	2.7	none	frequency<LOD	5,6,9, 10,33
Droplet digital PCR for HIV-1 DNA	coefficient of variation depends on template number (Strain et al., submitted); accuracy superior to kinetic PCR (5 fold at low copy numbers)	variable, 5×10^6^ cells in this study	resting CD4^+^ T cells or PBMC	primers: 2539→2562, 2659→2634. probe: 2589→2604	copies per 10^6^ cells	2	3.2	normalized using RNAse P to quantify host genomic DNA	frequency<LOD; primer mismatch	53
*Alu* PCR for integrated HIV-1 DNA	detects individual integrated HIV-1 genomes with standard curve to correct for sites too far from an *Alu* sequence to be detected; coefficient of variation = 0.20	variable, 5×10^6^ cells in this study	resting CD4^+^ T cells or PBMC	primers, outer: *Alu* primer, 800→782; inner: 525→542, 619-599. probe: 559→596	copies per 10^6^ cells	3	3.0	normalized by [DNA] assuming 1 µg = 150,000 cells	all assays above LOD in this study	39, 40, 55, 80
qPCR for HIV-1 DNA rectal biopsies	coefficient of variation for 10 copy standard = 0.18	up to 30 3 mm biopsies	cells from biopsy	primers: 522→543, 640→626. probe: 581→559.	HIV-1 genomes per 10^6^ CD4^+^ T cells	0.05	5.3	normalized by [DNA] and %CD4^+^ T cells (assuming 1 ug = 160,000 cells and all virus in CD4^+^ T cells)	all assays above LOD in this study	44
Droplet digital PCR for 2-LTR circles	coefficient of variation depends on template number (Strain et al., submitted); accuracy superior to kinetic PCR (>10 fold at low copy numbers)	variable, 5×10^6^ cells in this study	resting CD4^+^ T cells or PBMC	primers: 9588→9607, 48→28. probe: 556→530	copies per 10^6^ cells	2	1.1	normalized using RNAse P to quantify host genomic DNA	frequency<LOD; primer mismatch	53
Single copy assay for residual viremia	sensitivity superior to approved clinical assay; internal control for virus recovery	8 ml plasma	plasma	primers: 1299→1323	copies/ml of plasma	0.2	1.1	none	viral RNA<LOD; primer mismatch	46,47, 67

a HXB2 coordinates.

b Based in standard sample size. Except for residual viremia, LOD is expressed as infectious units or copies per 10^6^ cells. For residual viremia, the LOD is 0.2 copies/ml of plasma.

c Dynamic range is reported here as the difference in log units between the highest value measured in these study patients and the limit of detection of the relevant assay.

d Based on repeat measurements in the same patient as reported in reference 10 and assuming no decay in the reservoir.

**Table 3:** Cell types analyzed and viral species detected in assays.

	Cell types analyzed			Replication-competent viral species			Replication-defective viral species			
Assay	Resting CD4^+^ T cells	Activated CD4^+^ T cells	Monocyte/macrophage	Linear unintegrated HIV-1 DNA^a^	Integrated HIV-1 DNA	Plasma virus	Linear unintegrated HIV-1 DNA^a^	Integrated HIV-1 DNA	2-LTR circles	Plasma virus
Viral out-growth assay on resting CD4^+^ T cells	√			√	√					
Droplet digital PCR for HIV-1 DNA in PBMC	√	√	√	√	√		√	√	√	
Droplet digital PCR for HIV-1 DNA in resting CD4^+^ T cells	√			√	√		√	√	√	
*Alu* PCR for integrated HIV-1 DNA in PBMC	√	√	√		√			√		
*Alu* PCR for integrated HIV-1 DNA in resting CD4^+^ T cells	√				√			√		
qPCR for HIV-1 DNA in rectal CD4^+^ T cells	√	√	√^b^	√	√		√	√	√	
Droplet digital PCR for 2-LTR circles in PBMC	√	√	√						√	
Droplet digital PCR for 2-LTR circles in resting CD4+ T cells	√								√	
Single copy PCR assay on plasma virus						√				√

a This form is readily detected in viremic patients but is absent in patients on stable HAART (reference 35).

b Infected cells frequencies are normalized based on the fraction of CD4^+^ T cells present in the biopsy sample and assuming that all infected cells are CD4^+^ T cells.

### Viral outgrowth assay

**Figure 1:**
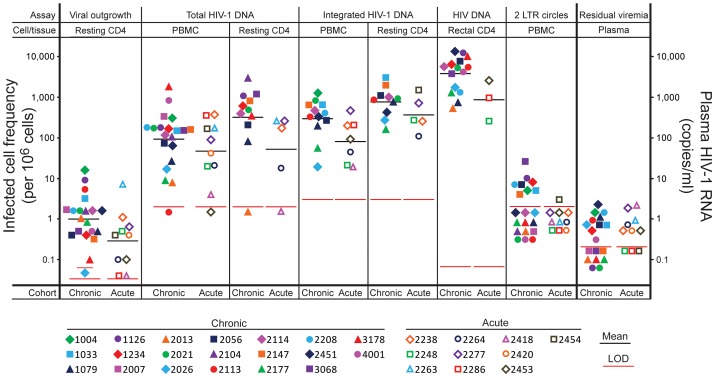
HIV-1 persistence assessed by six different assays performed on the indicated cell or tissue obtained from patients starting HAART during acute/early infection (open symbols) or chronic infection (closed symbols). Geometric mean values are indicated by a horizontal black line. Long red lines indicate the limit of detection (LOD) for the relevant assay with a standard input sample size. Short red lines indicate patient-specific limits of detection in cases where the blood or tissue sample was smaller than normal. Negative assays are indicated by samples plotted below the red LOD lines.

Replication-competent HIV-1 was isolated from purified resting CD4^+^ T cells from peripheral blood in 29/30 study participants (Figure 1). Infected cell frequencies showed a log normal distribution with a geometric mean frequency of 0.64 IUPM, consistent with previous reports [6], [9], [10], [52]. Latently infected cells were readily detected by this method in patients starting HAART during acute/early HIV-1 infection (mean = 0.28 IUPM). The mean frequency of latently infected cells was significantly lower in patients starting HAART in acute/early HIV-1 infection compared to those starting during chronic infection (0.28 vs. 0.97 IUPM, P = 0.048), although there was substantial overlap between the two populations (Figure 1). The frequency of latently infected cells was not correlated with the time between infection and the initiation of a suppressive HAART regimen (r = 0.18, P = 0.34), suggesting that the size of the reservoir does not increase continuously during untreated HIV-1 infection. For a single study participant, replication-competent virus was not detected even after repeat culture. Based on input cell number, the frequency of latently infected cells in this patient was <0.06 IUPM.

The limit of detection of this assay is determined by the input number of resting CD4^+^ T cells. With a 180 ml blood sample, the average yield of resting CD4^+^ T cells in millions was 28.3±14.7. With therapeutic strategies that reduce the reservoir by more than 1.5 logs, latently infected cells would no longer be detectable in the majority of patients unless larger blood volumes or leukopheresis samples were used (Figure 1).

### HIV-1 DNA in PBMC

A simple approach for quantifying persistent HIV-1 is to measure HIV-1 DNA using PCR in unfractionated PBMC. This was done using a droplet digital PCR approach that has greater accuracy than standard real time PCR methods, particularly with low template numbers [53]. HIV-1 DNA was detected in 28/30 PBMC samples (Figure 1). Values varied over a ∼2 log range with a geometric mean value of 74 copies/10^6^ PBMC for the entire cohort. Two subjects (2113 and 2453) had values that were below the limit of detection (2 copies/10^6^ PBMC). Interestingly, for these two subjects, cells with replication-competent virus were readily measured in the virus outgrowth assay (5.4 and 0.1 IUPM, respectively). HIV-1 DNA values were generally lower in subjects starting HAART in acute/early HIV-1 infection compared to those starting during chronic infection (geometric mean values 47 vs. 93 copies/10^6^; P = 0.30). The frequency of PBMC with HIV-1 DNA showed a modest but significant correlation with the time between infection and the initiation of a suppressive HAART regimen (r = 0.38, P = 0.037).

**Figure 2:**
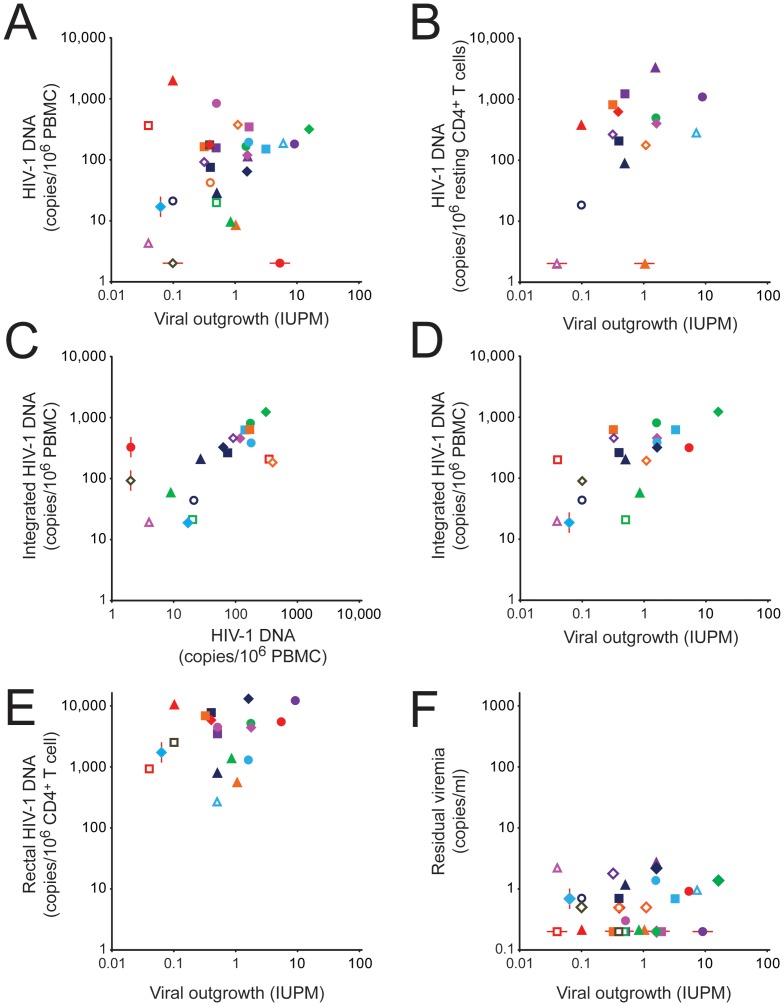
Correlation between assays for HIV-1-infected cells in patients on HAART. (**A**) Correlation between infected cell frequencies measured in the viral outgrowth assay on purified resting CD4^+^ T cells and the droplet digital PCR assay for HIV-1 DNA in unfractionated PBMC. (**B**) Correlation between the viral outgrowth assay on purified resting CD4^+^ T cells and the droplet digital PCR assay for HIV-1 DNA in purified resting CD4^+^ T cells. (**C**) Correlation between the droplet digital PCR assay for HIV-1 DNA and the *Alu* PCR assay for integrated HIV-1 DNA in PBMC. (**D**) Correlation between the viral outgrowth assay on purified resting CD4^+^ T cells and the *Alu* PCR assay for integrated HIV-1 DNA in PBMC. (**E**) Correlation between the viral outgrowth assay on purified resting CD4^+^ T cells from the blood and the PCR assay for HIV-1 DNA in rectal CD4^+^ T cells. (**F**) Correlation between the viral outgrowth assay on purified resting CD4^+^ T cells from the blood and the single copy assay for residual viremia. Patients starting HAART during acute/early or chronic infection are indicated by open or closed symbols, respectively. See Figure 1 for patient ID key. The red lines indicate samples that fell below the limit of detection of the relevant assay. These samples were not used in the calculation of the correlation coefficients.

To determine whether HIV-1 DNA levels in PBMC could be used as a surrogate measure of the size of the latent reservoir, we examined the correlation between results of the viral outgrowth assay on purified resting CD4^+^ T cells and the droplet digital PCR assay for HIV-1 DNA in PBMC from the same blood sample. As shown in Figure 2A and Table 4, there was essentially no correlation between the two assays (r = 0.20, P = 0.29) for the combined study population. Based on 95% confidence intervals for r, a strong correlation (r>0.6) could be excluded (Table 4). For patients treated during acute/early infection, a modest correlation that did not reach statistical significance was observed (r = 0.46, P = 0.18), but there was no correlation between the two assays for patients initiating HAART during chronic infection (r = −0.038, P = 0.87).

**Table 4:** Correlation of PCR-based assays with viral outgrowth assay.

Patient populations^a^:	Total				Acute				Chronic			
Assay	r^b^	95% CI^c^	P^d^	n	r^b^	95% CI^c^	P^d^	n	r^b^	95% CI^c^	P^d^	n
Droplet digital PCR for HIV-1 DNA in PBMC	0.20	−0.17 to 0.52	0.29	30	0.46	0.23 to 0.85	0.18	10	−0.038	−0.47 to 0.41	0.87	20
Droplet digital PCR for HIV-1 DNA in resting CD4^+^ T cells	0.45	−0.06 to 0.77	0.08	16					0.10	−0.53 to 0.66	0.76	11
*Alu* PCR for integrated HIV-1 DNA in PBMC	0.70	0.36 to 0.88	**0.0008**	19	0.25	−0.62 to 0.84	0.59	7	0.76	0.34 to 0.93	**0.0038**	12
*Alu* PCR for integrated HIV-1 DNA in resting CD4^+^ T cells	0.41	−0.13 to 0.76	0.13	15					0.38	−0.33 to 0.81	0.28	10
qPCR for HIV-1 DNA in rectal biopsy CD4^+^ T cells	0.26	−0.22 to 0.64	0.28	19					0.17	−0.35 to 0.62	0.53	16
qPCR for HIV-1 RNA in rectal CD4^+^ T cells	0.46	−0.01 to 0.76	0.053	18					0.38	−0.16 to 0.75	0.38	15
RNA/DNA ratio in rectal CD4^+^ T cells	0.57	0.14 to 0.82	**0.013**	18					0.52	0.01 to 0.81	**0.047**	15
Droplet digital PCR for 2-LTR circles in PBMC	0.19^e^	−0.18 to 0.52	0.31	30	0.12^e^	−0.55 to 0.69	0.75	10	0.06^e^	−0.39 to 0.49	0.81	20
Droplet digital PCR for 2-LTR circles in resting CD4^+^ T cells	0.38^e^	−0.15 to 0.74	0.15	16					0.037^e^	−0.57 to 0.62	0.91	11
Single copy PCR assay on plasma virus	0.070^f^	−0.30 to 0.42	0.71	30	−0.14^f^	−0.71 to 0.54	0.71	10	0.14^f^	−0.32 to 0.55	0.55	20

a Correlations were analyzed for the entire study population (n = 30) and for subpopulations of study subjects starting HAART during acute/early (n = 10) or chronic (n = 20) HIV-1 infection. Because of limitations in the size of blood or tissue samples, not all assays were performed for all subjects, and the n for each assay is shown in the last column. Correlation coefficients were only computed for n>5.

b Correlation coefficient for correlation with the viral outgrowth assay. Except where indicated, the Pearson correlation coefficient is shown.

c 95% confidence intervals for the correlation coefficient. Strong correlations were considered to be formally excluded when the upper limit of the 95% confidence interval was <0.6.

d P values <0.05 are indicated in bold type.

e Because of the large number of samples in which 2-LTR circles were below the limit of detection (2 copies/10^6^ cells), a Spearman's rank correlation coefficient was computed using a value of 1 copies/10^6^ cells for the samples in which 2 LTR circles were not detected.

f Because of the large number of plasma samples that had HIV-1 RNA levels below the limit of detection (0.2 copies/ml), a Spearman's rank correlation coefficient was computed using a value of 0.1 copies/ml for negative assays.

### HIV-1 DNA in purified resting CD4^+^ T cells

We next examined whether the correlation between the results of the viral outgrowth and PCR assays might be improved if HIV-1 DNA levels were measured in purified resting CD4^+^ T cells instead of unfractionated PBMC. HIV-1 DNA was detected in 14/16 samples (Figure 1). The geometric mean level of HIV-1 DNA was 186 copies/10^6^ resting CD4^+^ T cells. In two patients (#2013, 2418), levels were below the limit of detection (2 copies/10^6^ resting CD4^+^ T cells). For these two subjects, cells with replication-competent virus were readily measured in the virus outgrowth assay (1.05 and 0.04 IUPM, respectively). As a measure of infected cell frequency, the PCR assay gave substantially higher values than did the viral outgrowth assay performed on the same blood samples (186 vs. 0.62 infected cells/10^6^ resting CD4^+^ T cells, P<0.0001). This difference has been noted previously [5] and may reflect the high fraction of proviruses that are defective [41], [42].

Cells harboring defective viral genomes could accumulate over time during untreated disease. We did not, however, observed a significant correlation between the frequency of resting CD4^+^ T cells with HIV-1 DNA and the time between initial infection and suppression of viral replication on HAART (r = 0.33, P = 0.20), the time on a suppressive HAART regimen (r = 0.076, P = 0.78), or the total time since infection (r = 0.30, P = 0.26).

**Figure 3:**
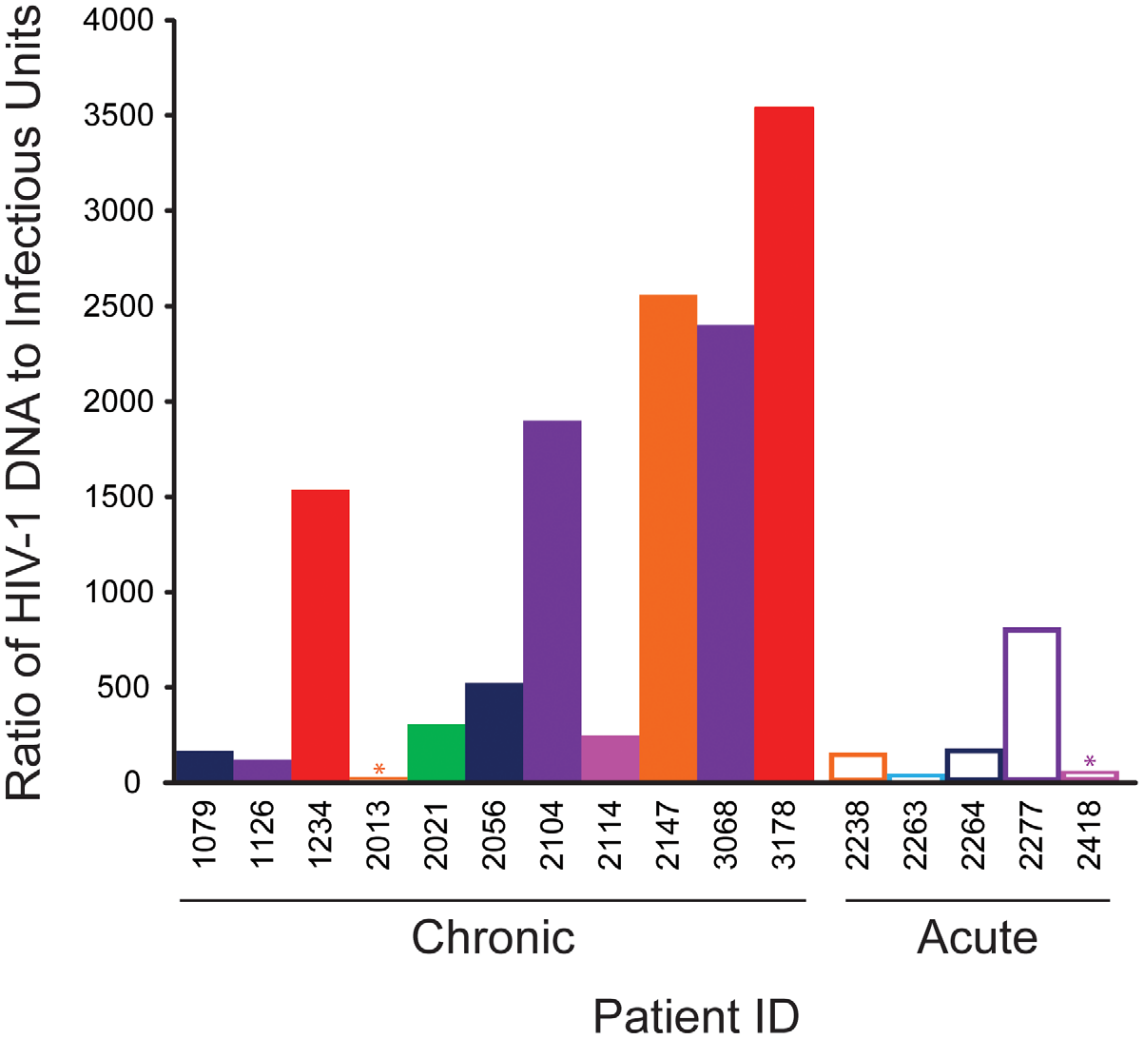
Ratio of infected cell frequencies determined by droplet digital PCR for HIV-1 DNA or by viral outgrowth assay. Analysis was done on the same sample of purified resting CD4^+^ T cells. * indicates maximum values in cases in which the HIV-1 DNA level was below the limit of detection (2 copies/ml).

If the proportion of defective proviruses was the same in different patients, then the measurement of HIV-1 DNA in resting CD4^+^ T cells from patients on HAART might provide a simpler surrogate measure of the latent reservoir. However, as is shown in Figure 3, the ratio of infected cells detected in the PCR vs. viral outgrowth assays is highly variable from patient to patient (range <2 to 3540) even when both assays are performed on the same sample of purified resting CD4^+^ T cells. A subset of patients who initiated therapy during chronic infection showed very high ratios (>1000∶1). For this reason, there is only a modest correlation between the results of the two assays for the combined population (Figure 2B and Table 4, r = 0.45, P = 0.08). In patients treated during chronic infection, no correlation is observed (r = 0.10, P = 0.76) The levels of HIV-1 DNA in unfractionated PBMC and in purified resting CD4^+^ T cells showed a strong correlation (r = 0.78, P = 0.0004). This reflects the fact that in patients on HAART, the stable reservoir for HIV-1 is located primarily in resting CD4^+^ T cells [6]. For most patients, infected cell frequencies were higher in resting CD4^+^ T cells than in PBMC (Figure 1). This is the expected result if most of the infected cells in the blood are resting CD4^+^ T cells. However, if substantial numbers of activated T cells and monocytes are infected, then the resting CD4^+^ T cell value may not be higher. Chun and colleagues have shown that even in patients on HAART, infected cells frequencies as measured by DNA PCR can be higher among activated than resting CD4^+^ T cells [54].

### Integrated HIV-1 DNA in PBMC and purified resting CD4^+^ T cells

Levels of integrated HIV-1 DNA were also measured in PBMC and purified resting CD4^+^ T cells from study participants using a previously described *Alu* PCR assay [39], [40], [55]. As shown in Figure 1, integrated HIV-1 DNA was detected in 19/19 PBMC samples, at a geometric mean frequency of 186 copies/10^6^ PBMC. The frequencies were significantly lower in patients starting HAART in acute/early vs. chronic infection (84 vs 286 copies/10^6^ PBMC, P = 0.04). As was observed with the droplet digital PCR assay for HIV-1 DNA, levels of integrated HIV-1 DNA were higher in purified resting CD4^+^ T cells than in unfractionated PBMC (geometric mean values 604 vs 186 copies/10^6^ cells, Figure 1). Also consistent with the results of the droplet digital PCR assay was the finding that the frequency of resting CD4^+^ T cells with integrated HIV-1 DNA was much higher than the frequency of latently infected resting CD4^+^ T cells detected in the viral outgrowth assay performed on the same sample (by 1000 fold). In paired samples, the mean infected cell frequencies were 604 vs. 0.61/10^6^ resting CD4^+^ T cells (P<0.0001).

Measurements of integrated HIV-1 DNA by *Alu* PCR and of total HIV-1 DNA by droplet digital PCR correlated well with each other both for samples of PBMC (Figure 2C, r = 0.63, P = 0.0042) and resting CD4^+^ T cells (r = 0.85, P = 0.0079). These results are consistent with the conclusion that in patients on long term HAART, most of the HIV-1 DNA is integrated, with unintegrated forms making only a minor contribution (see below) [38]. The fact that infected cell frequencies detected by *Alu* PCR were higher (186 vs 46 copies/10^6^ PBMC, P = 0.0003) may reflect differences in assay standardization. This assay provides definitive detection of integrated HIV-1 DNA through the use of an initial PCR with a primer in an *Alu* element and a primer in HIV-1. For each infected cell, the distance to the nearest *Alu* element is different [56]. This influences amplification efficiency, and some proviruses are located too far from an *Alu* element to be detected. To circumvent this problem, a correction is applied based on an integration standard containing proviruses integrated at different distances from the nearest *Alu* element [39], [40], [55]. The accuracy of the method depends on how closely the distribution of provirus-*Alu* element distances in the standard matches the distribution of distances in a particular patient sample. It is possible that the higher levels of integrated HIV-1 DNA detected in some patients result from over-correction for proviruses missed by *Alu*-PCR. Alternative explanations include underestimation of infected cell frequency by droplet digital PCR or issues with the normalization methods used in one or both assays.

Interestingly, there was a highly significant positive correlation between the level of integrated HIV-1 DNA in PBMC and the frequency of latently infected resting CD4^+^ T cells determined in the viral outgrowth assay on the same blood sample (Figure 2D and Table 4, r = 0.70, P = 0.0008). When only patients starting HAART during chronic infection were considered, the correlation remained significant (r = 0.76, P = 0.0038). However, when the *Alu* PCR and viral outgrowth assays were performed on the same sample of purified resting CD4^+^ T cells, the correlation was weaker and not statistically significant (r = 0.41, P = 0.13), possibly related to the small number of samples and small number of genomes assayed.

### HIV-1 DNA and RNA in rectal biopsy samples

Because the GALT is the site of very active viral replication in untreated patients with acute HIV-1 infection [57]–[61], we measured levels of HIV-1 DNA and RNA in the rectal biopsy samples from 19 study participants using qPCR. CD4^+^ T cells were enumerated in each sample by flow cytometry, and results were expressed as copies per CD4^+^ T cell. HIV-1 DNA was readily detectable in all samples (Figure 1). The geometric mean value for these 19 samples was 3015 copies/10^6^ CD4^+^ T cells. In paired comparisons, these values were significantly higher than the levels of infection of resting CD4^+^ T cells from the peripheral blood detected by digital droplet PCR (4282 vs. 263 copies/10^6^ cells, paired t-test: P<0.0001) or by *Alu* PCR (2977 vs. 600 copies/10^6^ cells, paired t-test: P = 0.0008). The level of HIV-1 DNA in rectal CD4^+^ T cells was not significantly correlated with the frequency of latently infected resting CD4^+^ T cells in blood as measured by the viral outgrowth assay (Figure 2E, r = 0.26, P = 0.28). However, a strong correlation could not be formally excluded based on 95% confidence intervals (r = −0.22 to 0.64, Table 4). There was a significant correlation between the level of HIV-1 DNA in rectal biopsy samples and the frequency of infected cells in the peripheral blood as measured by digital droplet PCR (r = 0.58, P = 0.015) or by *Alu* PCR (r = 0.65. P = 0.016).

HIV-1 RNA was also detected by qRT-PCR in all samples. The geometric mean level was 1985 copies/10^6^ rectal CD4^+^ T cells. These values cannot be used to establish infected cell frequencies because of the likely presence of multiple HIV-1 RNA molecules in some individual infected cells. Levels of HIV-1 RNA correlated well with measures of HIV-1 DNA in the same samples (r = 0.8811, P<0.0001). The geometric mean RNA/DNA ratio was 0.68. RNA/DNA ratios in rectal biopsies were significantly correlated with the frequency of resting CD4^+^ T cells in peripheral blood that scored in the viral outgrowth assay (r = 0.57. P = 0.013).

### 2-LTR circles

2-LTR circles represent abortive integration events. They have been used as a measure of recent infection in some studies, although controversy remains about their stability [62]–[66]. 2-LTR circles were measured in PBMC and purified resting CD4^+^ T cells from study subjects using droplet digital PCR (Figure 1). Circles were detected in only 9 of 30 PBMC samples. Among these the geometric mean level was 6.8 copies/10^6^ PBMC. This was 27 fold lower than the total level of HIV-1 DNA in the same samples measured by droplet digital PCR (162 copies/10^6^ PBMC, P<0.0001). As expected, levels of 2-LTR circles in purified resting CD4^+^ T cells from peripheral blood were higher than levels in unfractionated PBMC (geometric mean 13 copies/10^6^ resting CD4^+^ T cells), but again this level was much lower (by 34 fold) than the total level of HIV-1 DNA in the same samples measured by droplet digital PCR (467 copies/10^6^ resting CD4^+^ T cells, P<0.0001). These results demonstrate that 2-LTR circles make up only a small fraction of the total HIV-1 DNA measured by PCR in patients receiving on HAART. By Spearman's rank correlation analysis, levels of 2-LTR circles in PBMC or resting CD4^+^ T cells were not significantly correlated with infected cell frequencies measured in the viral outgrowth assay (rho = 0.19, P = 0.31 and rho = 0.38, P = 0.15 respectively).

### Single copy assay for residual viremia

Residual viremia was detectable in 20/30 study subjects, 13/20 in the chronic cohort and 7/10 in the acute cohort (Figure 1). Among the patients with detectable residual viremia, the geometric mean level was 0.78 copies/ml, consistent with previous studies [46], [47], [67]. There was no significant difference between the acute and chronic cohorts in the proportion of patients with detectable residual viremia (7/10 vs. 13/20) or in the level of detectable residual viremia (0.84 vs. 0.75 copies/ml, P = 0.75). Scatter plots show no obvious correlation between residual viremia and the viral outgrowth assay (Figure 2F). We considered the possibility that such a correlation could have been obscured by the failure to detect residual viremia in one third of the subjects. Negative results in the single copy assay could be due to primer mismatch or levels of residual viremia below the detection limit [47]. If patients for whom the single copy assay was negative are excluded, the correlation between residual viremia and the frequency of latently infected cells in the viral outgrowth assay is weak (r = 0.24, P = 0.33). If all patients are included in the analysis and a single copy assay value of 0.1 copies/ml is assumed for those patients with negative results in this assay, the rank correlation is also very weak (rho = 0.070, P = 0.71). Thus regardless of whether the negative values in the single copy assay represent primer mismatch or values below the limit of detection, it is difficult to construct a scenario where there is strong correlation between residual viremia and the viral outgrowth assay. A strong correlation could be formally excluded for the total population and for subpopulations starting HAART during acute/early or chronic infection. Residual viremia was not correlated with the level of infection in PBMC by droplet digital PCR (rho = −0.25, P = 0.18), the level of infection of CD4^+^ T cells in the rectal biopsies (rho = 0.12, P = 0.61), or the level of 2LTR circles (rho = −0.109, P = 0.57). Because the limit of detection of this assay with a sample volume of 8 ml is 0.2 copies/ml, a one log reduction in the size of viral reservoirs contributing to residual viremia would render values unmeasurable.

## Discussion

With the discovery of new agents that reactivate latent HIV-1, clinical trials of HIV-1 eradication strategies have begun [32]. No available clinical assay measures the size of the latent reservoir. Patients being enrolled in eradication studies have been on HAART for years and already have undetectable levels of viremia by standard clinical assays (detection limit 50 copies/ml). Therefore, there is an urgent need for laboratory assays to determine the efficacy of eradication strategies. Here we compare eleven different approaches for quantitating persistent HIV-1 in patients on HAART. The analysis involved seven analytical approaches and four different kinds of tissue samples. Assays were carried out using well characterized patients on long term stable HAART. Results were compared to the viral outgrowth assay that was originally used to define the latent reservoir [5], [6], [9], [10], [33].

We first evaluated PCR assays for HIV-1 DNA in unfractionated PBMC or resting CD4^+^ T cells. PCR quantitation of HIV-1 DNA in unfractionated PBMC perhaps offers the best chance for a scalable, high throughput assay for the latent reservoir. Using a novel droplet digital PCR assay, we detected HIV-1 DNA in PBMC from 28/30 subjects, with a mean infected cell frequency more than 100 fold higher than the mean frequency of resting CD4^+^ T cells that release replication-competent virus in the viral outgrowth assay. An even greater difference was observed when the viral outgrowth and droplet digital PCR assays were run on the same sample of purified resting CD4^+^ T cells. In part, this difference is likely to reflect the detection by PCR methods of cells harboring defective viral genomes such as cells with viral genomes that have been lethally hypermutated by APOBEC3G [42]. The difference between viral outgrowth and PCR-based assays was also observed in patients who start HAART in acute/early HIV-1 infection, suggesting that it is not simply the result of accumulation of cells with defective viruses over time. If a constant proportion of the infected resting CD4^+^ T cells contained defective genomes, then PCR measurements might provide a reliable surrogate measure of reservoir size in cross-sectional analysis. However, for the whole study population and the subset initiating HAART during chronic infection, a strong correlation (r>0.6) between results of the PCR assay for HIV-1 DNA and the viral outgrowth assay can be formally excluded. A larger sample size is needed to determine whether some correlation exists for patients initiating HAART early. It is possible that as the infection progresses, cells with defective viral sequences accumulate at different rates in different patients so that there is eventually very little correlation between the viral outgrowth assay and PCR-based assays. The ratio of infected cell frequencies determined by the two different assays varies by over 3 logs, with a subset of the patients who initiated therapy during chronic infection showing very high ratios. Taken together, these results are consistent with the hypothesis that differential accumulation of resting CD4^+^ T cells with defective viral sequences obscures the relationship between the frequency of cells detected in the virologic and molecular assays.

Most patients start therapy during chronic infection, and it is problematic that readily scalable PCR assays for total HIV-1 DNA on PBMC do not provide a precise reflection of the size of the latent reservoir, at least for cross-sectional analysis. It remains possible that PCR assays will be useful in following individual patients participating in eradication trials, but it is not yet clear that latency-reversing strategies will cause proportionate reductions in the latent reservoir and in the total pool of cells with HIV-1 DNA. For example, some cells with defective viral genomes may not express viral genes in response to latency-reversing agents. Hypermutated genomes typically have stop codons in every open reading frame [42], and thus cells carrying hypermutated genome may not be eliminated by latency reversing strategies that depend on viral cytopathic effects or CTL-mediated clearance. These cells might still be detected by PCR-based assays even when cells with replication-competent viral genomes were being eliminated.

Initial studies established that the latent reservoir in resting CD4^+^ T cells consists of cells with stably integrated viral genomes, [4], [5]. We therefore evaluated a well established *Alu* PCR assay specific for integrated viral genomes. Infected cell frequencies determined by this method were similar to and well correlated with frequencies determined by the droplet digital PCR assay, which does not distinguish integrated and unintegrated viral genomes (r = 0.85, P = 0.0079 for resting CD4^+^ T cells). These results are consistent with the notion that most of the HIV-1 DNA in resting CD4^+^ T cells of patients on HAART is integrated. Linear unintegrated forms, which are prevalent in untreated patients [35], are labile [37], [68] and are not seen in the absence of ongoing viral replication. Circular unintegrated forms (2-LTR circles), when detected, were present only at extremely low levels. Although levels of integrated HIV-1 DNA correlate well with measurements of HIV-1 DNA by the droplet digital PCR assay, both assays can detect defective as well as replication-competent proviruses. Among the approaches evaluated, analysis of integrated HIV-1 DNA in PBMC showed the best correlation with results of the viral outgrowth assay on purified resting CD4^+^ T cells (Table 4, r = 0.70, P = 0.0008). The correlation was weaker when integrated HIV-1 DNA was measured in purified resting CD4^+^ T cells.

Because the GALT provides a major site for HIV-1 replication [57]–[61], we also measured the HIV-1 DNA and RNA levels in rectal biopsy samples. When normalized for the number of CD4^+^ T cells present, the DNA PCR assay gave infected CD4^+^ T cell frequencies that were significantly higher than infected cell frequencies in the blood, consistent with previous studies [43], [44]. HIV-DNA levels in rectal biopsy samples showed a modest correlation with HIV-1 DNA levels in cells from the peripheral blood, but not with results of the viral outgrowth assay. Overall, these results highlight the potential importance of the GALT as an HIV-1 reservoir. However several critical questions remain. It is important to understand the fraction of CD4^+^ T cells in the GALT that can produce replication-competent virus and whether or not the virus is generally latent in these sites. The HIV-1 RNA/DNA ratios measured in these samples were generally <1, but these values are difficult to interpret because of uncertainty regarding the distribution of RNA molecules among infected cells. Interestingly, RNA/DNA ratios in rectal biopsy samples showed a statistically significant correlation with the viral outgrowth assay (r = 0.57, P = 0.013). This may reflect the fact that the RNA∶DNA ratio provides some indication of the number of infected cells that have the capacity to produce viral RNA.

As a measure of viral persistence, the single copy assay for residual viremia is of particular interest because it detects ongoing virus production in patients on suppressive HAART regimens. Residual viremia was only detectable in two thirds of study subjects and did not correlate with infected cell frequencies as measured by the viral outgrowth assay. A precise correlation might be expected if the activation of latently infected resting CD4^+^ T cells was the major source of residual viremia. However, recent studies [69]–[71] have show that in many patients the residual viremia is dominated by viral clones that are profoundly underrepresented in resting CD4^+^ T cells from the peripheral blood, suggesting an additional source of residual viremia.

Another important factor in evaluating assays for persistent HIV-1 is the dynamic range. Specifically, it is important to understand how much of a reduction in the reservoir would be measurable with each assay assuming reasonable sample volumes. Among the assays evaluated, the single copy assay for residual viremia has the lowest operating range. The viral outgrowth assay is already run on large blood volumes (180 ml), and its dynamic range cannot be extended much further. PCR-based assays perhaps offer the largest dynamic range, but suffer from the caveats discussed above.

Other studies have compared assays for persistent HIV-1, and the results differ to some extent from the findings presented here. In an early study, Anton et al. compared several measures of persistent HIV-1 infection in patients on HAART [72]. Although these investigators noted some modest correlations between the results of different culture and PCR-based assays, their findings cannot be readily compared to those of the present study because the culture assays were not run on purified resting CD4^+^ T cells and the PCR-based assays were not normalized for input CD4 cell number. Chun et al. reported a weak but statistically significant correlation (r = 0.29, P = 0.001) between linear values for residual viremia measured with an assay that has a detection limit of 20 copies/ml and the level of HIV-1 DNA in resting CD4^+^ T cells [73]. Murray et al. [74] observed an accumulation of cells with HIV-1 DNA during the first two years of untreated HIV-1 infection, consistent with the differences in infected cells frequencies between the acute and chronic cohorts in our study. Cells with defective proviruses may fail to express viral proteins and may therefore be protected from viral cytopathic effects and host CTL. The cells will thus have a chance to accumulate. This may happen at different rates in different patients, greatly complicating measurement of the reservoir by PCR-based approaches. Of note, no other study has shown a precise correlation between the results of the viral outgrowth assay and any simpler assay.

Overall, the results of this study suggest that no PCR-based assay provides a precise and internally consistent indication of the amount of replication-competent HIV-1 in resting CD4^+^ T cells. These findings raise the important issue of how to quantify decreases in the latent reservoir in future HIV-1 eradication trials. The fundamental problem is that infected cell frequencies determined by PCR-based assays are at least 2 logs higher than infected cell frequencies determined by the viral outgrowth assay. Much of this difference may be due to cells carrying defective proviruses, for example those that have been lethally hypermutated by APOBEC3G. These defective proviruses may not be eliminated by strategies designed to target latently infected cells. In this situation, successful clearance of latently infected cells might be masked by a larger and unchanging pool of cells with defective proviruses. While PCR-based assays may overestimate the size of the reservoir, the viral outgrowth assay provides only a minimal estimate of the frequency of cells harboring replication-competent virus. The assay conditions were carefully chosen to induce blast transformation in 100% of the input resting CD4^+^ T cells [6]. In this situation, the failure of an infected cell to produce replication-competent virus can be due to defects in the provirus such as APOBEC3G-induced hypermutation [42] or large internal deletions [41]. However, epigenetic silencing [75], transcriptional interference [76], [77], and other factors could also in principle prevent some proviruses without intrinsic defects from scoring in the viral out growth assay. Importantly, no other culture assay, including those that use alternative T cell activating stimuli [7], [8], has detected a higher frequency of cells with replication-competent virus. Nevertheless, the development of assays that precisely quantitate the number of the latently infected cells that have the potential to release replication-competent virus *in vivo* is an important goal in eradication research.

## Materials and Methods

### Patient cohorts

This study enrolled 30 patients from two well established cohorts at the University of California San Francisco (UCSF). All study subjects provided informed consent. Twenty patients were from the SCOPE cohort, an ongoing longitudinal study of ∼1500 HIV-1-infected and uninfected adults. Infected individuals in this cohort started HAART during chronic infection (>180 days from estimated date of infection). Subjects were seen and interviewed at four-month intervals to ascertain: (1) current medications, (2) medication adherence, (3) recent intercurrent illnesses, and (4) recent diagnoses or hospitalizations. Plasma HIV-1 RNA levels and routine T cell immunophenotyping were performed at each visit.

Ten patients were recruited from the OPTIONS cohort, an ongoing longitudinal study of adults enrolled within 8 months of HIV-1 infection. The evaluation of patients with possible acute infection included detailed HIV-1 testing and exposure history and the following laboratory studies: (1) a high-sensitivity assay for plasma HIV-1 RNA, (2) a standard HIV-1 antibody EIA with western blot confirmation if positive, and (3) a less-sensitive (detuned) antibody EIA (LS-EIA). Screened subjects met one or more of the following criteria to be defined as having primary or recent HIV-1 infection: (1) repeated plasma HIV-1 RNA >5,000 copies/ml combined with a negative or indeterminate HIV-1 antibody test; (2) seroconversion within 6 months of a documented negative HIV-1 antibody test or (3) a history compatible with primary HIV-1 infection (including no prior positive HIV-1 antibody tests) and laboratory testing consistent with recent infection on the “detuned” antibody EIA. Eligible subjects were followed approximately every 3 months.

Eligibility criteria for all patients enrolled in the present study included: (1) confirmed HIV-1 infection, (2) documented prior initiation of one of the Department of Health and Human Services recommended/alternative HAART regimens [78], (3) at least 36 months of continuous HAART at study entry with no regimen changes in the preceding 24 weeks, (4) maintenance of plasma HIV-1 RNA levels below the limit of detection of conventional assays for at least 36 months (intermittent isolated episodes of detectable low-level viremia were allowed), (5) most plasma HIV-1 RNA levels below the level of detection (<40 copies RNA/ml), and (6) documented CD4^+^ T-cell count above 350 cells/µl for preceding 24 weeks. We excluded subjects who (1) had recent hospitalization, (2) recent infection requiring systemic antibiotics, (3) recent vaccination or (4) exposure to any immunomodulatory drug (including maraviroc) in the preceding 16 weeks.

### Patient samples

All subjects who met entry criteria for screening and agreed to participate in the study had an initial structured interview, phlebotomy, and HIV-1 testing using the established SCOPE and OPTIONS infrastructure. Once eligibility for the large volume blood draw (220 ml) and gut biopsy procedures were determined, blood was collected in tubes containing acid-citrate-dextrose (ACD), and 180 ml of the sample were shipped overnight at ambient temperature to the Johns Hopkins School of Medicine where peripheral blood mononuclear cells (PBMC) and resting CD4^+^ T lymphocytes were isolated for quantitative studies of proviral DNA and replication competent virus. Extensive previous studies have shown that cells can be recovered with high viability for virus culture assays after overnight shipment [79]. The remaining peripheral blood (40 ml) was sent to the UCSF AIDS Specimen Bank for studies of the host response and additional virologic studies.

At John Hopkins, PBMC were isolated using density gradient centrifugation. Supernatant plasma was frozen at −80°C and sent on dry ice to Dr. Sarah Palmer of the Karolinska Institute in Stockholm, Sweden for the single copy assay for plasma HIV-1 RNA [46]. Aliquots of PBMC were frozen as cell pellets for analysis of total and integrated HIV-1 DNA. Resting CD4^+^ T cells were purified from PBMC by negative depletion using biotinylated antibodies and anti-biotin magnetic beads. Briefly, CD4^+^ T lymphocytes were first isolated from PBMC by removing unwanted cell populations (CD4^+^ T cell Isolation Kit II; Miltenyi). Non-CD4^+^ T cells were labeled first with a cocktail of biotin-conjugated monoclonal antibodies followed by incubation with anti-biotin-conjugated magnetic microbeads. Unwanted cells were then removed using a LS MACS Column with the MACS Separator magnet (Miltenyi). Activated CD4^+^ T lymphocytes were then removed from the total CD4^+^ T cell population by labeling unwanted cells with biotin-conjugated antibodies to CD25, CD69, and HLA-DR and anti-biotin microbeads (Miltenyi). Labeled cells were magnetically removed using MACS MS columns with the MACS separator. The resting CD4^+^ T cell population was typically 98–99% pure as assessed by FACS analysis. Latent HIV-1 in resting CD4^+^ T cells was quantitated using the viral outgrowth assay (see below). Aliquots of purified resting CD4^+^ T cells were frozen as cell pellets and sent along with aliquots of unfractionated PBMC to UCSD and the University of Pennsylvania for assays of total and integrated HIV-1 DNA. Details of individual assays are given below.

A subset of study subjects underwent a rectosigmoid biopsy at San Francisco General Hospital. Up to 30 3-mm biopsies were obtained 10–30 cm above the anus with a disposable biopsy forceps (3.3-mm outside diameter). Biopsy specimens were suspended in RPMI 1640 containing 10% fetal calf serum, piperacillin–tazobactam (500 µg/ml), and amphotericin B (1.25 µg/ml), and transported within 2–3 h to the UCSF Core Immunology Laboratory where the tissue was digested with collagenase and needle shearing (see below). Cells were counted and frozen as cell pellets until analyzed for HIV-1 DNA and RNA.

### Ethics statement

Blood and rectal biopsies from patients were obtained through protocols approved by the UCSF Committee on Human Research. For the virus culture assay, blood was obtained from healthy donors through a protocol approved by the Johns Hopkins University School of Medicine Internal Review Board #4. All study subjects provided written informed consent prior to participation in the study.

### Assays

#### Viral outgrowth assay

The principal assay used to define the latent reservoir in resting CD4^+^ T cells is a limiting dilution virus culture assay [5], [6], [9], [10], [33]. This assay was performed as previously described [33] except that purification of resting CD4^+^ T cells was carried out using the two step magnetic bead depletion protocol described above instead of bead depletion followed by flow sorting. Five-fold serial dilutions of highly purified resting CD4^+^ T cells were stimulated with the mitogen PHA and a ten fold excess of irradiated allogeneic PBMC. This stimulation induces global T cell activation which reverses latency at least in a fraction of cells carrying integrated HIV-1 genomes. The release of replication-competent virus was detected by adding CD4^+^ lymphoblasts from HIV-1-negative donors. The virus replicates in these cells and after 2–3 weeks of culture, supernatants contain sufficient levels of virus that an ELISA assay for HIV-1 p24 antigen can be used to detect positive wells. CD8^+^ T cells, which can suppress viral replication, were depleted from the lymphoblasts that were added to the culture assay. The frequency of HIV-1 infected cells was determined by a maximum likelihood method [33].

#### Digital droplet PCR for total HIV-1 DNA

Cellular DNA was extracted using a Qiagen DNA Blood Midi Kit, following the manufacturer's protocol. DNA was ethanol precipitated following elution to increase concentration. The DNA concentration was estimated from the A260/A280 absorptivity ratio using a NanoDrop 2000 spectrophotometer (Thermo Scientific). When the DNA concentration was below the desired concentration for emulsification, the concentration was increased by ethanol precipitation and re-suspension. Where specified, templates were thoroughly mixed with background human genomic DNA obtained by identical extraction methods from HIV-1 seronegative donors (“PBMC DNA”) or with sonicated salmon sperm DNA (Agilent Technologies). Extracted DNA was heated to 95°C for 10 minutes, then quenched on ice prior to digestion with the restriction enzyme BSAJ-I (New England Biolabs) at 60°C for 1 hour. Plasmids encoding the entire HIV-1 genome (pNL4-3, AIDS Reference Research Reagent Repository) or a 2-LTR junction (a kind gift from Dr. Kristine Yoder, Ohio State University) were used as standards. Primers to conserved regions of HIV-1 pol (HXB2 positions 2536-2662) and to the HIV-1 LTR (HXB2 positions 9585-51) were used. An RPP30 (RNAse P) primer/probe set was used for host genomic DNA quantification. Samples were diluted 10-fold and RPP30 was assayed without multiplexing. The PCR reaction mixture was loaded into the Bio-Rad QX-100 emulsification device, and droplets were formed following the manufacturer's instructions. The contents were transferred to a 96-well reaction plate and sealed with a pre-heated Eppendorf 96-well heat sealer for 2 seconds, as recommended by Bio-Rad. Total DNA was amplified separately in an Applied Biosystems GeneAmp 9700 thermal cycler. Each reaction consisted of a 20 µL solution containing 10 µL ddPCR Probe Supermix, 900 nM primers, 250 nM probe, and template DNA with the following cycling conditions: 10 minutes at 95°C, 40 cycles each consisting of a 30 second denaturation at 94°C followed by a 58°C extension for 60 seconds, and a final 10 minutes at 98°C. After cycling, droplets were analyzed immediately or stored at 4°C overnight and until analysis.

Raw fluorescence data for each well were exported from the manufacturer's software (Bio-Rad QuantaSoft v. 1.2) for analysis. Analysis of individual event data was performed using custom software written in Mathematica 8.0 (Wolfram Research). Droplets were classified as positive, negative or ambiguous following a custom algorithm that filters out potentially spurious events (Strain et al., manuscript submitted). Events that remained ambiguous after all data processing were excluded. The number of template copies per unit volume *μ* was estimated from the number of positive events *n* detected in the corresponding channel and the number of total accepted droplets *N* by maximum likelihood. The distribution of templates within a drop was assumed to follow a Poisson distribution, and the number of positive droplets was assumed to follow a binomial distribution. Confidence intervals were estimated under the same assumptions. The droplet size was assumed to be 0.91 nl, consistent with the instrument manufacturer's software. Template copies per sample were computed by averaging over all available replicate wells. Total cellular DNA input was measured by halving the estimated number of RPP30 copies, and copy numbers per diploid cell equivalent were computed as the ratio of template (pol or 2-LTR) copies per diploid cell. Statistical analyses were performed using Graph-Pad Prism 5.0 software (GraphPad Software, Inc.) and Mathematica 8.0.

#### Measurements of integrated HIV-1 in resting CD4^+^T cells and PBMC

Integrated HIV-1 DNA was measured in PBMC or purified resting CD4^+^ T cells using a previously described *Alu* PCR [39], [40], [55], [80]. This assay detects only integrated proviruses because it relies on an initial amplification in which one primer hybridizes with a conserved sequence in *Alu* elements which are present in ∼2,000,000 copies in the human genome. The *Alu* primer is paired with an HIV-1 *gag* primer in the first round of amplification. This *Alu*-*gag* amplification is then followed by a second amplification that targets a sequence in the HIV-1 LTR (R-U5). The level of HIV-1 integration is quantitated by comparing the detection signal to an integration standard curve that correlates cycle thresholds with integration standard copy number. The integration standard was especially produced to contain genomes with integration sites at a variety of distances from *Alu* sites, mimicking the pattern of integration seen in natural infection [80]. DNA was isolated from frozen PBMC and resting CD4^+^T cell pellets using the Gentra Puregene Cell Kit (Qiagen) following the manufacturer's instructions. DNA was then diluted to 2 ug/mL and assayed in 42 replicates for integrated HIV-1 proviruses as previously described [54]. The first step reaction was performed for 40 cycles at the following conditions: 95°C for 15 s, 50°C for 15 s and 72°C for 3 min 30 s. Simultaneously and on the same plate, 42 reactions with only the *gag* primer were also performed following the same conditions. The product from the first step reaction was then diluted 1∶2 into the 2^nd^ step master mix. The second step was performed on an ABI 7500 FAST instrument following the FAST protocol (95°C for 0∶03 and 60°C for 0∶30) for 50 cycles. The cycle threshold values are then used to calculate an integration value using a standard curve as described [55]. In samples with low levels of integrated HIV-1 DNA (with integration detectable in <30% of wells), the percent positive method for calculation was utilized. In these cases, a cutoff value was generated by subtracting two standard deviations from the average of the cycle threshold values for PCR reactions with only the *gag* primer present in the first step. Any wells with a cycle threshold less than this cutoff value were considered “positive” signals. The percent of positive signals is calculated by dividing the wells counted by the total number of wells assayed (containing both the *Alu* and *gag* primers). A standard curve [55] showing a linear relationship between the copies of integrated HIV-1 and the percent of positive wells was used to calculate copy number in several of these samples. Occasionally, samples had too little DNA to do 42 replicates.

#### Assays for HIV-1 DNA and RNA in GALT

Rectal biopsy samples were separated into single cells using a modification of a published method [44], [81], [82]. Briefly, biopsies were subjected to three rounds of collagenase digestion, mechanical disruption (by passing through a blunt 16 gauge needle), clarification (by passing through a 70 µm cell strainer), and washing. The three aliquots of strained and washed cells were then combined, counted, and aliquoted for flow cytometry and nucleic acid extraction. For extraction, cells were lysed using 1 ml of Qiazol reagent (Qiagen), followed by 15 min centrifugation at 12,000 g at 4°C. RNA was extracted from the aqueous layer using the miRNeasy Mini kit (Qiagen) with on-column DNAase treatment (Qiagen RNase-Free DNase Set) and eluted in 30 µl of RNase-free water. DNA was extracted from the organic layer using 0.5 ml of back extraction buffer (4 M guanidine thiocyanate, 50 mM Na citrate, and 1 M Trizma base), followed by precipitation with 500 µl of isopropanol, 2 washes with 75% ethanol, and resuspension in 100 µl of EB buffer (Qiagen). RNA and DNA concentrations were measured using a ND-1000 spectrophotometer (NanoDrop).

Three replicate samples of 500 ng of DNA from each donor were assayed for total HIV-1 DNA using a modification of a published real time Taqman PCR assay that uses primers and a probe from the LTR region and can detect a single copy of HIV-1 in brain tissue [4]. Primers were F522-43 Kumar (5′ GCC TCA ATA AAG CTT GCC TTG A 3′; HXB2 522–543) and R626-43 Kumar long (5′ GGG CGC CAC TGC TAG AGA 3′; 626–643). The probe, 5′ CCA GAG TCA CAC AAC AGA CGG GCA CA 3′, was dual-labeled with 6-FAM(5′) and Black Hole Quencher BHQ-1(3′). Reaction volume was 50 µl with 25 µl of 2× Gene Expression Master Mix (Applied Biosystems), 10 pmol of each primer, 10 pmol of probe, and 5 µl of DNA (100 ng/µl). Cycling conditions were: 50°C for 2 min, 95°C for 10 min, then cycles of 95°C for 15 s and 59°C for 1 min. External standards (10^5^ to 1) were prepared from DNA extracted from known numbers of 8E5 cells (NIH AIDS Reagent Program), each of which contains one integrated HIV-1 genome per cell.

HIV-1 DNA copy numbers were measured in triplicate and normalized to cellular input, as determined by DNA concentration (assuming 1 µg total DNA corresponds to 160,000 cells). To account for variation in the number of CD4^+^ T cells in different samples, results were further normalized by the percent of all cells that were CD45^+^CD3^+^CD4^+^ (by flow cytometry) and expressed as copies/10^6^ CD4^+^ T cells [44]. This normalization is based on the assumption that most of the HIV-1 DNA in rectal cells is in CD4^+^ T cells. This assumption is based on a recent analysis of sorted rectal cell populations that found ∼90% of the HIV-1 DNA to be in CD4+ T cells (S. Yukl, unpublished results).

Three replicates of up to 500 ng of RNA from each sample were assayed for total processive HIV-1 RNA transcripts using primers and probe from the LTR region (as above). Reaction volume was 50 µl with 25 µl of 2× One Step RNA-to-Ct mix (Applied Biosystems), 10 pmol of each primer, 10 pmol of probe, 1.25 µl of 40× RT (Applied Biosystems), and 5 µl of RNA (100 ng/µl). Cycling conditions were: 48°C for 20 min, 95°C for 5 min, then 60 cycles of 9°5C for 15 s and 59°C for 1 min. External standards (2.5 to 2.5×10^5^) of genomic HIV-1 RNA were prepared by extracting the RNA from lab stocks of NL4-3 virions (using the QIAmp Viral RNA Mini Kit) and then quantifying the RNA via replicate measurements using the Abbot Real Time assay.

HIV-1 RNA copy numbers were measured in triplicate and normalized to cellular input into the PCR, as determined by RNA concentration (assuming that 1 ng RNA correspond to 1000 cells [83]), which has been shown to correlate with levels of glyceraldehyde phosphate dehydrogenase (GAPDH) RNA [44]. As an alternative method of normalizing to cell numbers, all RNA samples were assayed for GAPDH in triplicate by qRT-PCR with the conditions above, except with 2.5 µl of 20× GAPDH primer/probe mix [Applied Biosystems] and 3 µl of RNA. To account for variation in the number of CD4^+^ T cells in different samples, HIV-1 RNA copy numbers were further normalized by the percent of all cells that were CD45^+^CD3^+^CD4^+^ [44].

#### Single copy assay for residual viremia

The concentration of HIV-1 RNA in plasma was determined as previously described [46], [47], [67]. To control for recovery of HIV-1, each plasma sample is spiked with 30,000 copies of an internal virion standard derived from an unrelated retrovirus, the replication competent avian sarcoma-leukosis retrovirus vector RCAS BP(A). For samples expected to have <50 copies of HIV-1 RNA/ml, 8 ml of plasma were used. Total RNA was extracted from the pellet using proteinase K and guanidinium isothiocyanate and used as a template for reverse transcription and real-time PCR of a small (79 bp), highly conserved, coding sequence in HIV-1 *gag.* Using this primer and probe set, HIV-1 RNA can be amplified from about 80% of those infected with HIV-1 subtype B. PCR products are detected using fluorescent probes. For each plasma sample, 3 independent reactions were run with HIV-1 *gag*-specific primers and two with RCAS BP(A) *gag*-specific primers. The quantity of HIV-1 RNA in the original plasma sample was calculated from a standard curve generated for each assay using serial dilutions of *in vitro* transcripts of the same region of *gag*. Control experiments have shown that the recovery of HIV-1 RNA and RCAS BP(A) RNA was comparable and independent of the original plasma volume used in the range of 1–8 ml.

### Statistical methods

Acute/early and chronic cohorts were compared using 2-tailed t tests for independent samples. Log transformed virologic data met the D'Agostino-Pearson test for Normal distribution, and correlations were performed on log transformed data. For DNA PCR measurements of cell associated HIV-1 genomes, we assumed that infected cells carried a single provirus [84] and expressed the results as the frequency of infected cells. For culture assay and HIV-1 DNA data, one or two samples were below the limit of detection of the relevant assays. For these, an imputed value representing the lower of the limit of detection or a value corresponding to 1 percentile of a log normal distribution fitted to the measured values was used in the calculation of the Pearson correlation coefficient. In the case of the single copy assay for HIV-1 RNA in plasma, for which one-third of the measurements were below the limit of detection (0.2 copies/ml), a low imputed value of 0.1 was used to calculate the Spearman rank correlation coefficient. Similar results were obtained when an imputed value of 0.01 was used. Data analysis was done using Microsoft Excel and MedCalc software.
